# Persistent Heart Failure Despite Medical Therapy Leading to a Diagnosis of Cardiac Amyloidosis

**DOI:** 10.7759/cureus.43547

**Published:** 2023-08-15

**Authors:** Diva Maraj, Sruthi Ramanan, Parth M Patel, Mumtaz Memon, Elise Hawes

**Affiliations:** 1 Internal Medicine, Henry Ford Health System, Jackson, USA; 2 Medicine, Michigan State University, Lansing, USA; 3 Cardiology, Henry Ford Health System, Jackson, USA; 4 Cardiac Imaging, Henry Ford Health System, Jackson, USA

**Keywords:** adult cardiology, cardiology, systemic senile amyloidosis, transthyretin amyloidosis, amyloidosis

## Abstract

Cardiac amyloidosis is restrictive cardiomyopathy, commonly classified as either light-chain amyloidosis (AL) or transthyretin amyloidosis (ATTR), which can be further subdivided into wild-type (systemic senile amyloidosis) and hereditary ATTR amyloidosis. Advanced-stage, silent, and clinically undiagnosed amyloidosis has a poor prognosis, with a survival rate of six months and up to five years. We present a 72-year-old female with a past medical history of heart failure, with preserved ejection fraction, atrial fibrillation, systemic lupus erythematosus (SLE), and stage 3b chronic kidney disease, who presented with persistent shortness of breath, lower extremity pitting edema, jugular venous distension, and dyspnea despite optimal medical therapy. The patient was diagnosed with preserved heart failure in the past and was on guideline-directed medical therapy for over five years with no history of cardiac disease in the family. The patient’s previous echocardiogram revealed an ejection fraction of 65%. In order to determine the etiology of the patient's cardiomyopathy, she underwent cardiac magnetic resonance imaging (CMR), monoclonal gammopathy testing, and a Technetium pyrophosphate (99mTc-PYP) scintigraphy, of which the latter two were unrevealing. The CMR revealed increased wall thickness and multiple segments of midmyocardial to subendocardial late gadolinium enhancement, suggestive of infiltrative disease. Due to inconclusive testing, the patient underwent an endomyocardial biopsy and was determined to have wild-type, systemic senile amyloidosis, which held a poor prognosis. The patient was started on tafamidis, a new Food and Drug Administration (FDA)-approved therapy for systemic senile amyloidosis, and was discharged on the new medication, with frequent follow-up visits scheduled. Current treatment guidelines for cardiac amyloidosis include loop diuretics and spironolactone. Medications such as beta-blockers, angiotensin-converting enzyme inhibitors, and calcium channel blockers are not clinically effective. There are currently new medications on the horizon, such as tafamidis, which stabilizes the transthyretin tetramer and reduces the formation of amyloid. This case highlighted that patients who have persistent symptoms of heart failure, despite guideline-directed medical therapy, and without a history of genetic cardiac conditions, may also have a diagnosis of cardiac amyloidosis. Cardiac amyloidosis is often misdiagnosed or diagnosed late in the disease course; therefore, there is a need for increasing awareness of early diagnosis and treatment, including new FDA-approved medications for a better chance of survival.

## Introduction

Cardiac amyloidosis is a rare but severe form of restrictive cardiomyopathy with a poor prognosis. There are two common types of cardiac amyloidosis: light-chain amyloidosis (AL) and wild-type transthyretin amyloidosis (ATTR) [1]. Patients with ATTR amyloidosis can present with heart failure symptoms, such as worsening dyspnea, shortness of breath, lower extremity edema, and arrhythmias. Patients are initially tested with echocardiography; however, more precise testing methods are also obtained. Cardiac magnetic resonance imaging (CMR) will determine if there is late gadolinium enhancement in the endomyocardium; however, its inconclusive serum protein electrophoresis can also be used [2,3]. Bone scintigraphy is also effective in diagnosing AL; however, the definitive diagnosis can be made with an endomyocardial biopsy, which will present with an apple-green birefringence pattern [4]. Patients can be treated with loop diuretics, such as furosemide, which is the mainstay of treatment [2]. Other treatments used for heart failure, such as beta-blockers, calcium channel blockers, and angiotensin-converting enzyme inhibitors, are rarely used, as they have not been shown to be effective clinically [2]. However, new Food and Drug Administration (FDA)-approved medications, such as tafamidis, can be used to treat ATTR amyloidosis [3]. This case highlights the need for routine surveillance in patients with heart failure for worsening symptoms, which may be indicative of amyloidosis.

## Case presentation

A 72-year-old woman, with a history of systemic lupus erythematosus (SLE), hypertension, hyperlipidemia, stage 3a chronic kidney disease, atrial fibrillation, diverticulitis, and heart failure of preserved ejection fraction of 56%, presented with worsening lower extremity edema and shortness of breath. She was previously admitted earlier in the year for congestive heart failure exacerbation, due to increased oxygen requirements and overt volume overload. Unfortunately, she still had residual edema and worsening shortness of breath. At presentation, the patient was in atrial fibrillation and later spontaneously converted to normal sinus rhythm. The patient was placed on 3 L of oxygen and had tachycardia, crackles bilaterally, and 3+ lower extremity pitting edema up to her sacrum. Her brain natriuretic peptide was elevated at 21,000, with a persistently elevated troponin of 199 on admission. The patient was started on loop diuretics, furosemide, 80 mg intravenous, twice a day.

**Figure 1:**
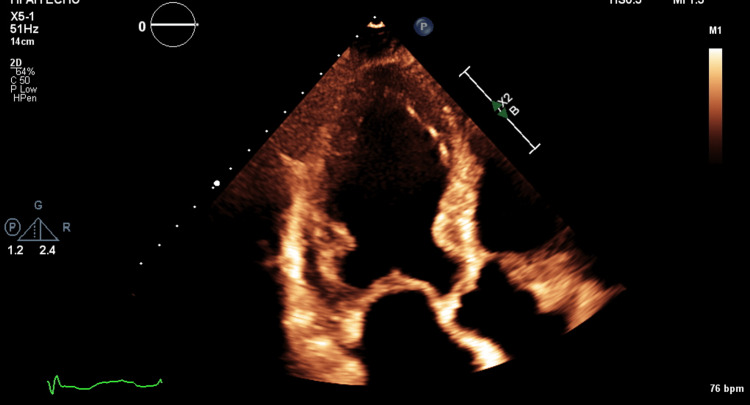
Left ventricular wall thickening. The basal and mid inferior wall and inferior septum appear worse than the rest.

**Figure 2:**
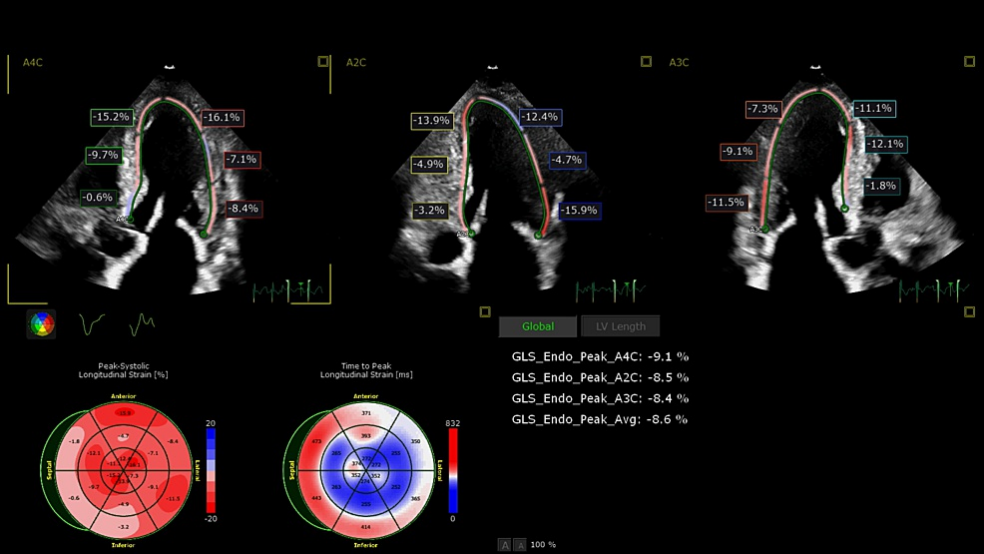
Echocardiogram results indicating left ventricular strain associated with amyloidosis and sub-clinical left ventricular dysfunction.

Given the persistent symptoms despite optimal medical therapy, the patient was further worked up with a right heart catheterization, which revealed mildly elevated right atrium pressure with no pulmonary hypertension noted. During diuresis, the patient's creatinine function did remain stable. The patient then underwent CMR, which revealed a left ventricular ejection fraction of 52%, with midmyocardial to subendocardial late gadolinium enhancement (LGE) in the basal-mid inferior, inferoseptal, and inferolateral segments. There was also increased wall thickness and multiple segments of midmyocardial to subendocardial LGE suggestive of infiltrative disease, as seen in Figures 1-2. Diagnostics included serum protein electrophoresis, which was unrevealing. Due to unrevealing lab work, the patient also underwent a bone scintigraphy scan, which was not conclusive for ATTR. The patient was then referred for an endomyocardial biopsy, which was positive for ATTR cardiac amyloidosis, with a positive birefringence pattern.

The patient was started on tafamidis, a newly approved therapeutic for wild-type systemic senile amyloidosis. Tafamidis stabilizes the transthyretin (TTR) tetramer and reduces the formation of TTR amyloid in the endocardium. The patient tolerated the medication well; however, she was deemed to have a poor prognosis with an expected survival of six months to one year.

## Discussion

Cardiac amyloidosis is a severe form of cardiomyopathy in delayed stages, caused by either a deposition of TTR or immunoglobulin light chains [1]. ATTR amyloidosis is caused by misfolding and deposition of TTR, which is made by the liver to transport thyroid hormone and retinol (vitamin A) [1]. It can be further subdivided into two types: light-chain systemic amyloidosis and wild-type ATTR amyloidosis, previously known as senile systemic amyloidosis, which is caused by the deposition of misfolded wild-type TTR [1,2]. ATTR amyloidosis is commonly diagnosed in older adults with heart failure of preserved ejection fraction and severe aortic stenosis [1]. Patients with TTR cardiac amyloidosis typically present at age ≥60 years and most commonly >70 years old [5]. Various TTR mutations are associated with differing ages of onset (ranging from 30 to 70 years) and differing risks of cardiomyopathy. Patients can present with a wide range of symptoms, including dyspnea, lower extremity edema, elevated jugular venous pressure, hepatic congestion, and ascites [1]. They can also have changes on electrocardiogram, such as left bundle branch block and atrial fibrillation [3,4].

If amyloidosis is suspected, the initial diagnostic test is echocardiogram [1]. Further evaluation is based on the patient's clinical symptoms, for patients with unexplained left ventricular hypertrophy, aortic stenosis with features associated with cardiac amyloidosis, or heart failure with symptoms or signs typical of amyloidosis. The next step is a CMR [1,2]. The three progressive LGE patterns identified in cardiac amyloidosis include none, subendocardial, and transmural, which correlate with the degree of myocardial infiltration [3,4]. Serum protein electrophoresis can also be completed, and if monoclonal protein is identified, along with echocardiographic or CMR findings consistent with cardiac amyloidosis, it suggests light-chain cardiac amyloidosis [1-3]. If monoclonal proteins are not found, the next step is bone scintigraphy, and the presence of grade 2- or 3-positive bone tracer on cardiac scintigraphy in a patient without monoclonal protein is highly specific for ATTR cardiac amyloidosis, and there is no need for a tissue biopsy [3]. An endomyocardial biopsy can be used to definitively diagnose ATTR amyloidosis, which will reveal apple-green birefringence using Congo red stain; however, it can have a poor yield due to patchy uptake [5,6].

Loop diuretics are the mainstay of treatment of decompensated heart failure, and standard guideline-directed medical therapy does not have the expected response in improvement in cardiomyopathy caused by amyloidosis [5]. Medications that lower the heart rate, such as beta blockers and CCB, are contraindicated as the cardiac output depends on the heart, rate and since the heart is infiltrated with amyloid fibrils, it does not respond to the mechanism of either beta-blockade or CCB [6].

Patients with ATTR cardiomyopathy with a New York Heart Association functional class of I-III can be treated with tafamidis [6]. A randomized control trial showed that it reduced mortality and cardiovascular-related hospitalizations and reduced the decline in quality of life [6]. Tafamidis stabilizes the TTR tetramer and reduces the formation of TTR amyloid. The current FDA-approved dose is 80 mg daily. There are also currently ongoing active trials for new medications for ATTR amyloidosis. These include Patisiran, which is a small anti-TTR interfering ribonucleic acid that interferes with hepatic TTR synthesis [6]. Inotersen is another treatment currently undergoing clinical trials, which is an antisense oligonucleotide that inhibits the hepatic production of TTR [6].

## Conclusions

This case highlights the need for routine surveillance in patients with heart failure and worsening clinical progression despite being on optimal medication therapy. These patients may unknowingly have a diagnosis of cardiac amyloidosis. Unfortunately, patients who have ATTR amyloidosis have a poor prognosis; however, with the development of new clinical drugs on the horizon, such as the FDA-approved medication, tafamidis, there is hope for a better prognosis in the future.
